# Risk Prediction of One-Year Mortality in Patients with Cardiac Arrhythmias Using Random Survival Forest

**DOI:** 10.1155/2015/303250

**Published:** 2015-08-25

**Authors:** Fen Miao, Yun-Peng Cai, Yu-Xiao Zhang, Ye Li, Yuan-Ting Zhang

**Affiliations:** ^1^Key Laboratory for Health Informatics of the Chinese Academy of Sciences (HICAS), Shenzhen Institutes of Advanced Technology, Shenzhen 518055, China; ^2^Shenzhen College of Advanced Technology, University of Chinese Academy of Sciences, Beijing 100049, China; ^3^Department of Senile Cardiovascular Medicine, The General Hospital of the People's Liberation Army, Beijing 100853, China; ^4^Joint Research Centre for Biomedical Engineering, Department of Electronic Engineering, Chinese University of Hong Kong, Shatin 00852, Hong Kong

## Abstract

Existing models for predicting mortality based on traditional Cox proportional hazard approach (CPH) often have low prediction accuracy. This paper aims to develop a clinical risk model with good accuracy for predicting 1-year mortality in cardiac arrhythmias patients using random survival forest (RSF), a robust approach for survival analysis. 10,488 cardiac arrhythmias patients available in the public MIMIC II clinical database were investigated, with 3,452 deaths occurring within 1-year followups. Forty risk factors including demographics and clinical and laboratory information and antiarrhythmic agents were analyzed as potential predictors of all-cause mortality. RSF was adopted to build a comprehensive survival model and a simplified risk model composed of 14 top risk factors. The built comprehensive model achieved a prediction accuracy of 0.81 measured by c-statistic with 10-fold cross validation. The simplified risk model also achieved a good accuracy of 0.799. Both results outperformed traditional CPH (which achieved a c-statistic of 0.733 for the comprehensive model and 0.718 for the simplified model). Moreover, various factors are observed to have nonlinear impact on cardiac arrhythmias prognosis. As a result, RSF based model which took nonlinearity into account significantly outperformed traditional Cox proportional hazard model and has great potential to be a more effective approach for survival analysis.

## 1. Introduction

Cardiac arrhythmias are defined as a group of conditions in which the electrical activity of the heart is irregular or faster or slower than normal [1]. Some arrhythmias are life-threatening and would result in sudden cardiac death if not treated in time. It is one of the most common causes of death when travelling to a hospital. A major challenge in the management of arrhythmias in hospital is the availability of reliable prognostic models that enable patients and physicians to have a realistic expectation of prognosis and to guide treatment options including medical treatment, use of devices, more intense monitoring, or end-of-life care. In addition, getting insights into which factors relate to poor outcome may help the physicians adopt appropriate medical treatments.

Until now, several models for predicting different kinds of cardiovascular diseases outcome such as heart failure (HF) and coronary heart diseases have been developed using data from clinical trials or observational studies [2–6]. In addition, several risk models for mortality in community were reviewed by Kwok et al. in [7]. However, researches on morality prediction for cardiac arrhythmias patients are still very rare as presented by Hinkle Jr. et al. [8]. In addition, most risk models presented above are based on multivariable Cox proportional hazard regression (CPH), which was proposed by Cox [9]. CPH is an intuitive and popular survival model by illustrating the importance of each variable and its relationship with a regression coefficient. However, proportional methods suffer from high variance and poor performance as demonstrated by Breiman [10, 11] as solving the model is very complex, especially for those involving multiple variables and further more nonlinear effects cannot be modeled. Fox example, considerable controversy is still unsettled regarding the precise association of body mass index (BMI) with prognosis. Even though BMI is often considered with poor survival in general population, some researchers such as Uretsky et al. have identified a possible obesity paradox among patients with heart disease in which increased body mass predicts better survival using univariate CPH [12]. The above results are biased due to a linear assumption between BMI and mortality and not considering the interaction between BMI and some other factors. Therefore, complex patterns about possible reverse causation in underweight individuals, including interactions with smoking and an unclear inflection point at which increasing body mass leads to increased risk, were noted by Adams, Flegal, and Fontaine et al. [13–15] through manually adding the interactions between BMI and other factors or subdivision of the population into different small groups. However, all of the methods mentioned above are from a subjective point of view. Random survival forests (RSF) modeling, a direct extension of random forest for survival analysis, is proposed by Ishwaran et al. [16] to handle the above difficulties by automatically assessing the complex effects and interactions among all variables from objective view, that is, following the inherent relationship between any factors and the predictive result. Ishwaran et al. also demonstrated that RSF has another advantage of insensitivity to noise brought by missing values or error data [16]. Thus, it has been used in several risk models for different kinds of diseases such as heart failure [17] by Hsich et al. and breast cancer [18] by Omurlu et al. The results show that the RSF model can identify complex interactions among multiple variables and performed better than traditional CPH model. Therefore, the aim of our study is to identify important risk factors and their complex effects on mortality and develop an available mortality risk model in cardiac arrhythmias patients using RSF.

## 2. Material and Methods

### 2.1. Study Population

Our study is based on the public MIMIC II (Multi-Parameter Intelligent Monitoring in Intensive Care) clinical database [19, 20], which contains comprehensive clinical data including results of laboratory tests, medications, ICD9 diagnoses, and more obtained from hospital medical information systems, for 32,536 ICU patients. The data were collected over a seven-year period, beginning in 2001, from a variety of ICUs (medical, surgical, coronary care, and neonatal), in Boston's Beth Israel Deaconess Medical Center (BIDMC). We defined the patients with cardiac arrhythmias according to ninth revision of the international classification of diseases (ICD9) adopted in the database with ICD9 of 427. We define the start point of each patient as the time of ICU admission and the end point as the death time in the hospital or 365 days after the start point. Using this approach, 10,488 patients with cardiac arrhythmias were extracted to establish the predictive model, during which 3,452 deaths occurred in hospital or after discharge over 1-year follow-up period for each patient.

### 2.2. Study Variables

Potential clinical variables previously reported to be associated with mortality were evaluated in our study. The following 40 variables were assessed for prognostic value: demographics including age, sex, and BMI, clinical variables such as arrhythmias type (CA, VF, VT, AF, and other slow arrhythmias), valvular heart diseases, renal failure, and CHF, laboratory variables with missing value less than 20%, including glucose, NA, K, SCR, BUN, RBC, WBC, PT, PTT, INR, BR, AST, ALT, and CKPK, and antiarrhythmic agents including class I, class II, class III, class IV, and class V agents (as listed in Table 1). All of the clinical variables are binary and defined as 1 if the patient suffers from this disease at the admission time, otherwise defined as 0. The laboratory variables are real-valued numbers measured at the admission time to the ICU and the measurement scales for all variables are presented in Table 1. The medication was defined as 1 if the patient was prescribed with and took this kind of medications during the ICU stay.

### 2.3. Statistical Analysis with Random Survival Forest

In our study, continuous variables such as age, BMI, SCR, and BUN were log-transformed before analysis to eliminate the tail effect brought by larger or smaller value. Random survival forest [16], a new extension of random forest for survival analysis, was implemented in our study to establish prediction models in the following ways:

(1) B bootstrap samples are randomly extracted from the original dataset, with each bootstrap sample precluding an average 37% of the data, that is, out-of-bag data (OOB data). B is defined as 1000 in our study.
(2) A survival tree is grown for each bootstrap sample to develop a comprehensive model composed of all 40 variables. 6 candidate variables are randomly selected for each node based on the rule defined in [16]. The candidate variable with the ability of maximizing the survival difference between child nodes is selected to split the node.
(3) Every tree was grown to full size until each leaf node is with less than *d*_0_ > 0 unique deaths. In our study, *d*_0_ is set to be 3.
(4) Select predictive variables by filtering on the basis of minimal depth, that is, the minimum distance from the trunk to the branch level of the nearest maximum subtree, which is the largest subtree whose root node splits on the variable. The smaller the minimal depth is, the more impact the variable has on prediction.
(5) Construct a simplified survival forest of 1000 survival trees based on the predictive variables selected above to get an optimized model with fewer variables.
(6) The cumulative hazard function for each terminal node in a grown tree is estimated by Nelson-Aalen estimator [21]. Drop each case *X*_*i*_ in the validation dataset down the grown tree; its cumulative hazard function is the Nelson-Aalen estimator [21] for the case's falling terminal node. The individual hazards are then averaged to compute the cumulative hazard function for each tree. The ensemble cumulative hazard function is then obtained by averaging the cumulative hazard functions for all of the grown trees. Nelson-Aalen estimator was also used for comparison between estimated and real cumulative hazard functions for the validation dataset.
(7) Prediction error is calculated based on Harrell c-statistics [22] for the ensemble cumulative hazard function, with the *b*th value being the error rate for the ensemble computed using the first *b* trees. The method to calculate variable importance (VIMP) for a variable *x* is presented in [16].

To demonstrate the effectiveness of the RSF based models, Cox proportional hazards models were then used for comparing and evaluating the basic association between potential risk factors and mortality [23], with Wald tests for significance testing. Cross validation [24] was employed to validate the proposed models. In *k*-fold cross validation, the original dataset is randomly divided into *k* equal-size subsets with a single subset as the validation dataset and the remaining *k* − 1 subsets as the training datasets. The cross validation process is repeated *k* times. The results from the *k* processes are averaged to produce a single estimation. As 10-fold cross validation is commonly used in most of situations, it was also used in our study to optimize the prediction performance.

All analyses were carried out with R version 3.0.1. RSF was implemented based on the “RandomSurvivalForest” package, which can be accessed from the public R software packages freely.

### 2.4. Baseline Characteristics of the Study Cohort

The baseline characteristics of alive and dead patients one year after presenting cardiac arrhythmias of the study cohort are shown in Table 1. During a followup of 1 year for each patient, 3452 individuals died, while 7036 were censored. The mean age for those who died within one year was 75.32 ± 12.84 and 71.94 ± 23.6 for patients that remained alive. From Table 1 we can see that demographics and clinical risk factors such as age, BMI, CA, slow arrhythmias, CHF, and stroke were significantly different between alive and dead patients. All laboratory results were also significantly different between alive and dead patients. With the exception of class V agents, all antiarrhythmic agents were significantly different between alive and dead patients.

## 3. Results

### 3.1. Model Validation

To validate the performance of proposed risk models, prediction ability in terms of c-statistic with 10-fold cross validation was used in our study. The c-statistics for different models (comprehensive model versus simplified model) with different methods (RSF versus CPH) are presented in Table 2. From the table we can see that the RSF can improve the discrimination ability greatly for both comprehensive model and simplified model with high significance level (c-statistic of 0.81 versus 0.733 for comprehensive model and 0.799 versus 0.718 for simplified model with 10-fold cross validation). In addition, the proposed simplified model can realize good predictive accuracy with limited variables based on a c-statistic of 0.799.

### 3.2. Comprehensive Risk Model Predicting 1-Year Mortality with RSF

**Figure 1:**
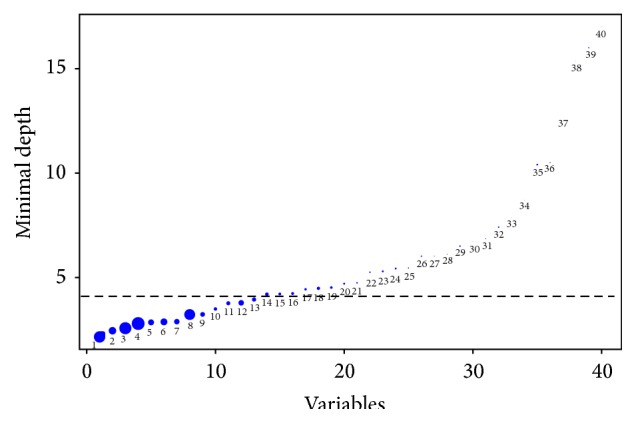
Minimal depth from RSF analysis. Horizontal line is threshold for separating predictive variables that are below the line. The diameter of each circle is in proportion to the forest-averaged number of maximal subtrees for that variable: 1: cardiac arrest, 2: log of BUN, 3: log of BMI, 4: log of AST, 5: log of age, 6: log of SCR, 7: log of BR, 8: log of K, 9: log of WBC, 10: log of ALT, 11: log of NA, 12: log of CKPK, 13: class II agents, 14: log of glucose, 15: log of INR, 16: CHF, 17: renal failure, 18: log of RBC, 19: log of PTT, 20: class V agents, 21: log of PT, 22: stroke, 23: sex, 24: AF, 25: class IV agents, 26: myocardial infarction, 27: hypertension, 28: uncomplicated diabetes, 29: valvular heart disease, 30: slow arrhythmias, 31: VT, 32: VF, 33: hypothyroidism, 34: complicated diabetes, 35: class III agents, 36: liver disease, 37: chronic pulmonary heart disease, 38: acute pulmonary heart disease, 39: class I agents, 40: bundle branch block.

From the comprehensive RSF analysis with all 40 variables, 14 variables were selected to be predictive for 1-year mortality, including cardiac arrest, BUN, BMI, AST, age, SCR, BR, K, WBC, ALT, NA, CKPK, class II agents, and glucose (the detailed minimal depths of all variables can be seen from Figure 1, in which 14 predictive variables were separated from the remaining nonpredictive variables by the horizontal line). The 6 variables on the extreme left including cardiac arrest, BUN, BMI, AST, age, and SCR are easily seen to be the most predictive variables.

### 3.3. Simplified Model Development for Clinical Application

**Figure 2:**
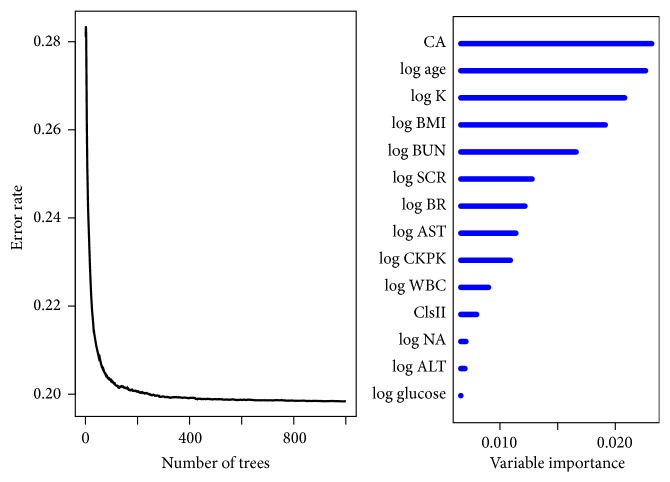
Error rates with simplified RSF for ensemble cumulative hazard function and VIMP for predictors. ClsII indicates class II agents. log*∗* indicates log of index.

**Figure 3:**
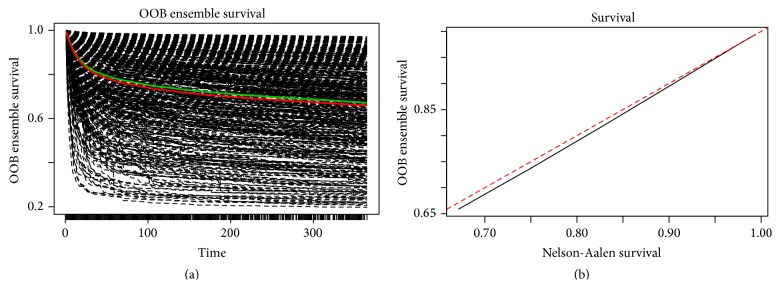
(a) Ensemble survival function for each individual. Red line is overall ensemble survival, while green line is Nelson-Aalen estimator. (b) Comparison of the population ensemble survival function and the Nelson-Aalen estimator.

In order to improve the availability of the proposed model, we reduced the comprehensive models to include the most important 14 risk factors selected from the comprehensive RSF analysis and developed a simplified model. The error rates for ensemble cumulative hazard function and VIMP for predictors are presented in Figure 2 with an estimated c-statistic of 0.799 (the detailed method for calculating prediction error and VIMP is presented in Section 2).  Figure 3 gives the correlation between ensemble survival function and nonparametric Nelson-Aalen estimator, which is an alternative estimator for Kaplan-Meier. From the figure we can see that the ensemble survival function is very close to the curve with Nelson-Aalen estimator (*r* = 0.999, *p* < 0.001). In other words, the estimated survival function using RSF basically conforms to the real survival curve.

### 3.4. Risk Model Predicting 1-Year Mortality with CPH

As presented in Section 2.3, we also use traditional CPH for comparison to demonstrate the effectiveness of the proposed RSF model. After multivariable CPH analysis with all 40 predictors, the following 23 risk factors presented in Table 3 were found to be independent significant predictors for mortality, during which the first 6 important predictors with highest absolute BC coefficient were decreasing K, decreasing BMI, cardiac arrest, class III agents, increasing age, and BUN. The predictive accuracy of the model was reasonable, with a c-statistic of 0.733 using 10-fold cross validation. These variables are a bit different from RSF based model as the RSF identified the nonlinear effect of the continuous variables on the mortality, which will be discussed in Section 4.

## 4. Discussion

### 4.1. Complex Patterns and Interactions Identified Using RSF

**Figure 4:**
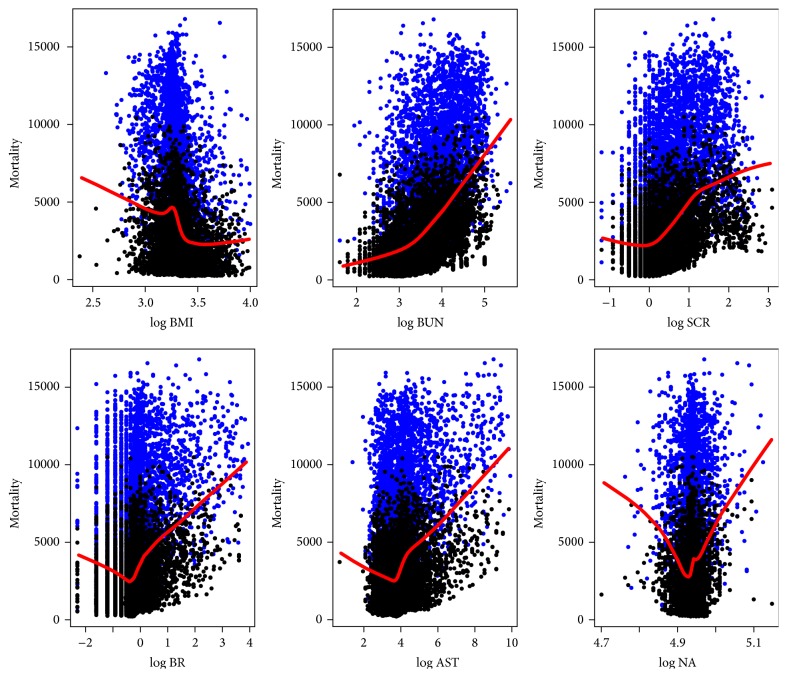
Ensemble mortality against given continuous variables. Mortality is presented in terms of total death number. Points colored with blue correspond to events, while black ones correspond to censored observations. log*∗* indicates log of index.

**Figure 5:**
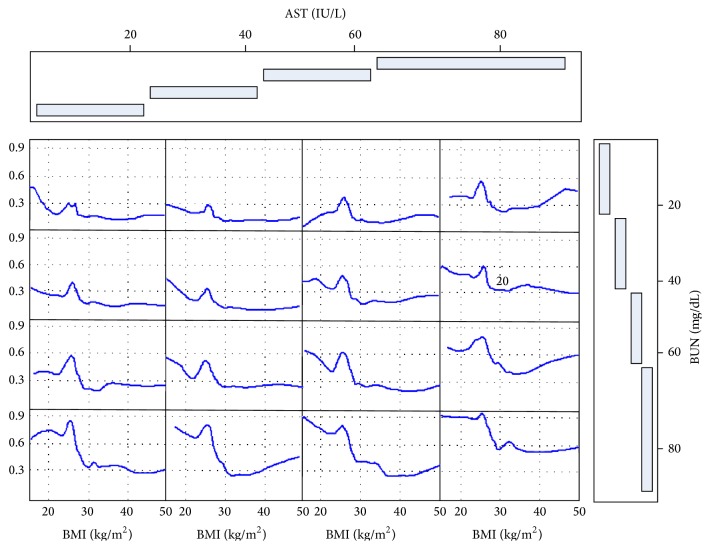
RSF-estimated 1-year mortality as a function of BUN, AST, and BMI. Smoothed curves are computed based on the estimated mortality for each patient.

**Figure 6:**
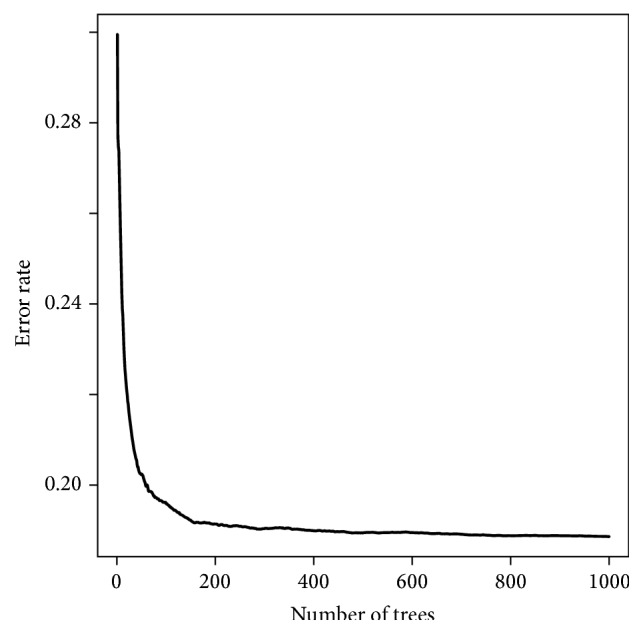
Estimated error rate with comprehensive RSF for different grown trees.

Figure 4 shows the marginal effect of a given continuous variable on the 1-year mortality from RSF analysis. From the figure we can see that the association between the continuous variables like BMI, BR, AST, SCR, and NA and 1-year mortality is nonlinear.  Figure 5 displays how the RSF model shows interaction among the 3 most important continuous variables including BUN, AST, and BMI and 1-year predicted mortality. Patients with the highest BUN and AST have the largest mortality and most have low BMI. One-year predicted mortality increased sharply with the elevation of BUN, with about 15% for those with a BUN below 20 mg/dL and nearly 50% for those with a BUN above 58 mg/dL. The mortality rate depends more on AST than BMI for patients with lower BUN, while more on BMI for those with higher BUN. The above mentioned interactions and nonlinear relationships were not prespecified by the analyst but identified by the forest. That is why the RSF has a better discrimination ability than CPH with the error rate (1-c-statistic) of 19% (the details about estimated error rate according to different grown trees can be seen from Figure 6).

From the above analysis we can see that RSF has the advantage of automatically identifying the nonlinear effect and complex interactions among multiple variables, using plots such as those shown in Figures 4 and 5. Therefore, RSF can improve the accuracy greatly compared to standard methodologies (c-statistic of 0.81 versus 0.733 for comprehensive model and 0.799 versus 0.718 for simplified model using 10-fold cross validation), especially for a model with many continuous variables that have nonlinear effects on the predictive result. However, RSF is limited in identifying predictors with small number of population, that is, class III agents, due to its insensibility of noise. Therefore, a more comprehensive survival model will be developed in the future.

### 4.2. Comprehensive Risk Model versus Simplified Model

The comprehensive risk model, which is built with demographics and clinical and laboratory risk factors and antiarrhythmic agents, has advantage in discrimination ability with c-statistic of 0.81 using RSF and 0.733 using CPH. However, too many variables would influence the efficiency and result of the fitting process using mathematical models and thus lead to an unstable performance for the prediction model. Therefore, an optimized model that can balance the discrimination ability and cost is very important for its availability. The proposed simplified model can be used to derive a prognostic score and to estimate the risk of death with good accuracy (c-statistic of 0.799 with 10-fold cross validation) using only 14 risk factors and thus has great potential to be an optimized risk model in a real-life cohort of ICU patients with cardiac arrhythmias.

### 4.3. Risk Factors for Mortality in Patients with Cardiac Arrhythmias

RSF identified cardiac arrest, BUN, BMI, AST, age, SCR, BR, K, WBC, ALT, NA, CKPK, class II agents, and glucose as the 14 predictive factors of survival in the cohort of 10,488 ICU patients with cardiac arrhythmias. These variables are a bit different from what was found in traditional Cox proportional hazard model analysis, by which SCR and NA are not significant predictors. In fact, both predictors exact nonlinear effects on mortality from the RSF analysis.

For predictors including BR, AST, SCR, and NA, both smaller and larger values for the variables would induce high mortality and the same with other continuous predictors such as ALT, K, and CKPK. It can be easily understood that there is a normal range for every laboratory test, too high and too low mean different kinds of abnormal status. For example, normal serum NA levels are between approximately 135 and 145 mEq/L. A serum level of less than 135 mEq/L is generally defined as hyponatremia while it is defined as hypernatremia for more than 145 mEq/L. It is demonstrated [25] that the lower the NA level, the higher the risk for the mortality. Values above 180 mEq/L are demonstrated to be associated with a high mortality rate, particularly in adults [26]. In addition, we found that the mortality would increase sharply with the elevation of BUN. It was also demonstrated that elevation BUN is independent predictive for long-term mortality [27] and subsequent mortality in patients with acute coronary syndromes [28].

Predictor BMI exacts complex impact on cardiac arrhythmias mortality. From its marginal effect on mortality presented in Figure 4, we can find that mortality decreases in a tiny amplitude with an elevation of BMI at lower BMI, and then with a sharp increase while close to an approximate point of 25 kg/m^2^, after which mortality begins to sharply decrease. It was also demonstrated [29] that, compared to normal weight (BMI 18–24.9), underweight (BMI < 18) was associated with increased risk of death while overweight (BMI 25–29.9) leads to significantly decreased risk. Interestingly, this pattern is highly dependent on BUN levels from their interaction analysis (as presented in Figure 5). For BUN values smaller than 20 mg/dL, significantly healthy renal function, the inflection point pattern is much straighter (subplots on the extreme top side) and becomes sharper and sharper with the elevation of BUN level (subplots from top to bottom). These results validate and add strength to the findings by Ishwaran et al. [16] using a more important renal function index BUN rather than creatinine clearance that the relationship of low BMI with high mortality depends on renal function, and this phenomenon disappeared while the renal function improved. In other words, high mortality rate in patients with low BMI may not due to underweight, but due to the weight loss accompanied with renal disease.

During five arrhythmias types, cardiac arrest is identified as the most important predictor leading to mortality with both methods. It is well known that unexpected cardiac arrest can lead to death within minutes: this is called sudden cardiac death (SCD). It was also demonstrated in [30] that sudden cardiac arrest exacts a significant mortality with a survival rate of only 2%. However, ventricular fibrillation, which is considered as one of the most emergency arrhythmias and responsible for nearly 50% of all causes of out-of-hospital cardiac arrests, is not identified as a predictive variable in our models, perhaps because moderate ventricular fibrillation is reversible if treated in time for ICU patients. It is also demonstrated [31] that if patients with in hospital VF were defibrillated early the survival could be very high.

During five classes of antiarrhythmic agents, class II agents are identified as the most prevalent medications in arrhythmias patients (nearly 75%) and most effective on prolonging survival times. Even though class I agents are not identified as predictive in RSF analysis, they would reduce survival rate in a certain degree from CPH based model. This finding validates the results reported by Echt et al. [32], in which class I agents were found to increase mortality instead of lowering it after myocardial infarction. That is why class I agents are least used medications (approximately 2% in the database).

## 5. Conclusion

In this paper, we developed a prognostic model that combines demographics and clinical and laboratory risk factors and antiarrhythmic agents for predicting the mortality of ICU patients with cardiac arrhythmias using random survival forest. The RSF model showed a much better separation of 1-year survivors and nonsurvivors with a c-statistic of 0.81 using 10-fold cross validation, compared with 0.733 using conventional CPH. In addition, a simplified model was built based on the most important 14 risk factors and thus presented to be more applicable in real-life cohort, showing good separation with c-statistic of 0.799 with RSF and 0.718 with CPH. Moreover, several variables that exact nonlinear effects on mortality and with interaction were automatically identified with RSF. Therefore, RSF has been demonstrated to be a potential effective approach for survival analysis.

The major limitation of our study is that we have not validated our models in external cohorts; nevertheless, the replicability of the result should be sufficient with 10-fold cross validation method. In addition, due to incomplete data, some potential predictors, such as triglyceride (TG) and total cholesterol (TC), were not included in the proposed model. More comprehensive model can be developed by adding these potential predictors with potentially better discrimination performance.

## Figures and Tables

**Table 1:** Baseline characteristics of dead and alive patients during one-year followup.

Characteristics	Dead	Alive	*P* value
*N*	3452	7036	
Demographics			
Age, years	75.32 (12.84)	71.94 (23.596)	<0.001
Gender, male	59%	57%	0.1
BMI, kg/m^2^	22.76 (102)	33.48 (2588)	<0.001
Clinical variables			
Arrhythmias type			
CA	518 (15%)	352 (5%)	<0.001
VF	138 (4%)	211 (3%)	0.459
VT	390 (11%)	844 (12%)	0.095
AF	2727 (79%)	5488 (78%)	0.281
Slow arrhythmias	276 (8%)	1055 (15%)	<0.001
HF	1484 (43%)	2110 (30%)	<0.001
Myocardial infraction	621 (18%)	1266 (18%)	0.867
Bundle branch block	18 (0.5%)	38 (0.6%)	0.168
Valvular heart diseases	517 (15%)	915 (13%)	0.088
Stroke	424 (12%)	633 (9%)	0.01
Hypertension	932 (27%)	2251 (32%)	<0.001
Acute pulmonary heart disease	79 (2%)	141 (2%)	0.238
Chronic pulmonary heart disease	178 (5%)	352 (5%)	0.277
Uncomplicated diabetes	690 (20%)	1547 (22%)	0.001
Complicated diabetes	242 (7%)	352 (5%)	0.001
Hypothyroidism	345 (10%)	704 (10%)	0.143
Renal failure	483 (14%)	422 (6%)	<0.001
Liver disease	138 (4%)	141 (2%)	<0.001
Laboratory variables			
K, mEq/L	4.81 (0.89)	4.96 (0.94)	<0.001
NA, mEq/L	139.21 (4.25)	138.85 (2.93)	<0.001
WBC, K/*μ*L	19.79 (13.88)	16.1 (8.13)	<0.001
RBC, K/*μ*L	4.09 (0.75)	4.15 (0.58)	<0.001
ALT, IU/L	185.57 (659)	87.54 (372)	<0.001
AST, IU/L	345.31 (1388)	125.49 (631)	<0.001
CKPK, IU/L	711.67 (4944)	532.99 (2061)	0.017
SCR, mg/dL	2.49 (2.03)	1.8 (1.76)	<0.001
BUN, mg/dL	56.64 (34.15)	38.68 (26.22)	<0.001
Glucose, mg/dL	196.91 (85.41)	187.57 (58.71)	<0.001
PT, seconds	22.97 (15.88)	20.78 (12.69)	<0.001
INR	3.29 (5.49)	2.49 (2.88)	<0.001
PTT, seconds	75.79 (47.99)	69.10 (43.64)	<0.001
BR, mg/dL	2.08 (4.66)	1.09 (2.02)	<0.001
Medications			
Class I agents	86 (2.4%)	112 (1.6%)	0.006
Class II agents	2481 (72%)	5366 (76%)	<0.001
Class III agents	153 (4.4%)	781 (11%)	<0.001
Class IV agents	810 (23%)	1205 (17%)	<0.001
Class V agents	2255 (65%)	4622 (66%)	0.716

**Table 2:** *C*-statistics for comprehensive model and simplified model with different methods.

Model	Method		
	RSF	CPH	*P* value
Comprehensive model	0.810	0.733	<0.01
Simplified model	0.799	0.718	

**Table 3:** Cox proportional hazard model with comprehensive risk factors.

Predictors	Coefficient	*P* value	HR	95.0% CI	
				Lower	Upper
Demographics					
log age	**.633**	**<0.001**	**1.883**	**1.637**	**2.167**
log BMI	**−.979**	**<0.001**	**.376**	**.313**	**.451**
Clinical risk factors					
Cardiac arrest	**.890**	**<0.001**	**2.435**	**2.164**	**2.741**
Slow arrhythmias	−.420	<0.001	0.657	0.572	0.755
CHF	.119	0.002	1.126	1.042	1.216
Myocardial infraction	.168	0.001	1.182	1.069	1.307
Stroke	.340	<0.001	1.405	1.266	1.560
Renal failure	.243	<0.001	1.275	1.132	1.437
Laboratory risk factors					
log K	**−1.146**	**<0.001**	**.318**	**.256**	**.395**
log WBC	.266	<0.001	1.305	1.210	1.408
log RBC	−.443	0.001	.642	.494	.834
log BUN	**.562**	**<0.001**	**1.754**	**1.605**	**1.916**
log glucose	.187	0.002	1.205	1.071	1.356
log CKPK	−.106	<0.001	.900	.872	.928
log AST	.379	<0.001	1.460	1.357	1.572
log ALT	−.255	<0.001	.775	.722	.832
log PT	−.447	<0.001	.640	.523	.782
log INR	.334	<0.001	1.397	1.228	1.590
log BR	.107	<0.001	1.113	1.062	1.165
Medications					
Class I agents	0.376	0.002	1.456	1.147	1.849
Class II agents	−0.316	<0.001	0.729	0.670	0.793
Class III agents	**−0.864**	**<0.001**	**0.421**	**0.352**	**0.505**
Class V agents	−0.203	<0.001	0.816	0.754	0.883

HR: hazard ratio; CI: confidence level; log *∗* indicates log of variables.

## List of Abbreviations

CA: Cardiac arrest
VF: Ventricular fibrillation
AF: Atrial fibrillation
VT: Ventricular tachycardia
K: Potassium [mEq/L] in blood
NA: Sodium [mEq/L] in blood
WBC: Leukocytes [K/*μ*L] in blood
RBC: Erythrocytes [m/*μ*L] in blood
SCR: Creatinine [mg/dL] in serum or plasma
BUN: Urea nitrogen [mg/dL] in serum or plasma
CKPK: Creatine kinase [IU/L] in serum or plasma
AST: Aspartate aminotransferase [IU/L] in serum or plasma
ALT: Alanine aminotransferase [IU/L] in serum or plasma
PT: Prothrombin time (seconds) in blood by coagulation assay
PTT: Activated partial thromboplastin time (seconds) in blood by coagulation assay
INR: International normalized ratio in blood by coagulation assay
Glucose: Glucose [mg/dL] in blood
BR: Total bilirubin [mg/dL] in serum or plasma
BMI: Body mass index (kg/m^2^)
CHF: Congestive heart failure
RSF: Random survival forest
CPH: Cox proportional hazard model.
